# Health literacy and attitudes toward clinical AI: a cross-sectional study in Istanbul

**DOI:** 10.3389/fdgth.2026.1771286

**Published:** 2026-05-07

**Authors:** Kadriye Sönmez

**Affiliations:** Department of Medical Documentation and Secretarial Program, Istanbul Topkapı University, Plato Vocational School, Istanbul, Türkiye

**Keywords:** artificial intelligence, digital health, health literacy, human-AI interaction, public attitudes, technology acceptance

## Abstract

**Objective:**

To examine whether health literacy is associated with attitudes toward clinical artificial intelligence (AI) and to describe subgroup differences in this association.

**Methods:**

We used two samples. A validation sample (*N* = 293) was used to develop and test the psychometrics of the Artificial Intelligence Knowledge, Usage, and Attitudes Scale (AI-KUAS). A separate application sample (*N* = 354) completed the 14-item Turkish Health Literacy Scale (HLS-14-TR) and the AI-KUAS. Analyses included correlations, group comparisons (t-tests/ANOVA with appropriate *post-hoc* procedures), and multiple linear regression adjusting for age, gender, and education. Two-tailed *α* = .05.

**Results:**

In the validation sample, a four-factor, second-order structure of the AI-KUAS was supported (CFI = .955, TLI = .943, SRMR = .037, RMSEA = .092), with high internal consistency (*α* = .961); a three-factor alternative was inferior (*Δ*CFI = .015; higher RMSEA). In the application sample, health literacy correlated moderately with AI attitudes (r = .51, *p* < .001) and remained a significant independent predictor in adjusted models (*β* = .39–.50, *p* < .001). Education showed additional positive associations with several subscales, whereas age and gender were not significant predictors. Model R^2^ values ranged from .17 to .31 across outcomes.

**Conclusion:**

Health literacy was positively and independently associated with more favorable attitudes toward clinical AI. Psychometric results suggested that perceived benefit and intention to use were closely related yet distinct dimensions within a four-factor structure. The findings align with literacy-aligned explanations, clear disclosure of AI use, and visible human oversight as practical implementation levers. Given the cross-sectional design and self-reported outcomes, longitudinal studies and measurement-invariance testing are warranted to support stronger causal and cross-group inference in future work.

## Introduction

Health literacy is considered a fundamental concept that defines an individual's capacity to access, understand, evaluate, and apply health information, and is recognised as a significant structure that determines the quality of the relationship established with health services (1, 2). This structure, which encompasses functional, interactive, and critical levels, represents the entirety of cognitive processes that enable the accurate interpretation of health information from the perspective of content and context. Recent studies pointing to inconsistencies between conceptual definitions and measurement tools show that the impact of health literacy on behavioural outcomes has a multidimensional basis (3). Considering that health information is progressively being provided by means of digital platforms, this fundamental structure paves the way for a competence area that is being reshaped in digital environments (4, 5).

Digital health literacy is defined as a subfield comprising skills specific to the digital context, such as accessing online health information, evaluating information, and managing privacy and data security (6, 7). This field serves as an extension of health literacy that expands through digital interaction and plays a decisive role in online information search and evaluation processes. Self-report-based measurement tools capture perceived competencies, while performance-based tools provide outputs that are more proximate to actual skills; eHEALS focuses on online information search skills, while DHLI (Digital Health Literacy Instrument) covers a more comprehensive set of digital competencies (8–10). The various processes for accessing and evaluating information in digital environments emerge as a significant factor determining the quality of users’ relationship with digital health services (11). However, despite extensive work linking both general and digital health literacy to navigation and appraisal of online health information, no empirical study has examined how these competencies relate to attitudes toward AI-mediated information in clinical settings, leaving an important conceptual and empirical gap.

Patterns observed in interactions with digital health services provide an explanatory framework for understanding the evaluation processes of individuals encountering AI-based systems in clinical settings. Findings regarding the frequency of use of patient portals, awareness levels, and information trust indicate that digital health literacy determines interaction with technology (12, 13). The fact that the impact of portal use on care quality or satisfaction varies contextually suggests that digital interaction is shaped by user competencies (14). The variable diagnostic accuracy of symptom screeners serves as a striking example highlighting the significance of the capacity for critical evaluation of digital outputs (15). The cognitive processes emerging in interactions with digital services create a parallel ground for understanding and confidently using clinical AI outputs from the perspective of (16).

In shaping attitudes towards clinical AI, tolerable reasons such as uncertainties encountered by users, information asymmetry, and the desire to be involved in decision-making processes form a significant starting point. The need for meaning, level of trust, and perception of accountability are central to user evaluations and these three elements become the fundamental determinants of the general trend towards clinical AI. Expectations regarding the presence of human oversight, the requirement for explainability, and transparency regarding the possibility of error are seen as key components supporting the formation of trust (17–21). Findings indicating that trust is shaped by cognitive processes in conjunction with health and digital literacy reveal that trust is formed by means of reliability signals and perceptions of who is responsible (22, 23). The emphasis on human-centred evaluation approaches in explainable AI literature supports the decisive role of literacy-based fundamental cognitive processes in understanding clinical AI outputs (24, 25). Given the complexity of the information generated by AI technologies in clinical settings, the necessity of a multidimensional evaluation approach to explain user attitudes becomes more apparent (17, 26).

The aim of the study is to examine how health literacy levels relate to attitudes toward clinical AI using a multidimensional approach. The analyses aim to clarify the ways in which cognitive and perceptual processes that evaluate clinical AI encounters connect with health literacy. In accordance with this aim, individuals’ attitudes toward clinical AI use, their trust in information generated by clinical AI, perceived benefits, and ethical evaluations were quantitatively assessed.

## Methods

### Study design and reporting

A cross-sectional observational design with a psychometric validation component was employed. Reporting adhered to STROBE guidelines for observational studies and established best practices in scale development and validation (27). Ethical approval was granted by the Istanbul Topkapi University Scientific Research and Publication Ethics Committee (E-49846378-050.04-2500013751; approval date 03 July 2025; meeting No. 2025/18, 01 July 2025). Written informed consent was obtained from all participants, and all procedures followed the Declaration of Helsinki and its amendments.

### Setting and participants

These districts were chosen to capture İstanbul's socioeconomic and demographic heterogeneity. Kadıköy represents a high-literacy, high-income urban population; Beşiktaş reflects central metropolitan areas with high digital access; and Bağcılar represents lower-income, high-density neighborhoods with more variable levels of health and digital literacy. Together, these areas reflect distinct segments of İstanbul's 15.9-million population base and allow recruitment across diverse educational and occupational groups. Using a multistage non-probability approach, districts were purposively selected to reflect socioeconomic diversity, and adults (≥18 years) residing in İstanbul who were able to read and respond in Turkish were recruited through convenience (intercept) sampling in accessible community settings. Participants were approached in community centers, public squares, and educational venues following local administrative permissions, and surveys were administered using paper-based forms or supervised tablet entry depending on site conditions. Participants who provided informed consent and completed the questionnaire were included. Psychometric analyses were conducted on participants with complete responses to the AI-KUAS items, and regression analyses used listwise deletion for missing values in any model variables. On this basis, the target sample size for the validation phase was defined *a priori* to ensure adequate precision for factor-analytic model testing.

The validation sample size (*N* = 293) was set *a priori* based on established recommendations for factor-analytic models and simulation-based evidence. Classical guidance suggests a minimum ratio of 10–15 participants per item, implying a requirement of approximately 150–225 participants for a 15-item scale. Monte Carlo simulation studies for ordinal CFA further indicate that stable parameter estimation, reliable standard errors, and convergence of WLSMV estimators typically occur with samples ≥250 under moderate-to-high factor loadings. Accordingly, a minimum target of 280 participants was set *a priori* to ensure adequate power for evaluating both first-order and higher-order structures. A supplementary *a priori* power analysis conducted in G*Power 3.1 indicated that detecting small-to-moderate effects (r = .20–.25; f^2^ ≈.04–.06) at *α* = .05 requires 240–310 participants to achieve.80–.90 power. The achieved sample size exceeded this threshold, meeting psychometric standards for robust model testing. The application sample (*N* = 354) additionally provided >.90 power to detect small-to-moderate associations between health literacy and AI attitude outcomes in adjusted analyses.

## Measures

### Health literacy

Health literacy was measured using the 14-item Turkish Health Literacy Scale (HLS-14-TR), previously validated in adult Turkish samples with acceptable internal consistency and factorial validity (28). Items index the ability to access, understand, appraise, and apply health information. Responses used Likert-type anchors; higher scores indicate higher literacy. Negatively keyed items (if any) were reverse-coded. Scale scores were calculated when ≥75% of items were answered.

### Attitudes toward clinical AI

Attitudes toward clinical AI were assessed with the Artificial Intelligence Knowledge, Usage, and Attitudes Scale (AI-KUAS), which was developed by the authors specifically for this study (English version provided as Supplementary File S1). The scale is a 15-item self-report instrument organized into four theorized subscales: Perceived Benefit (Items 1–4), Intention to Use (Items 5–8), Trust in Information (Items 9–11), and Ethical Concern (Items 13–15). Item 12 was excluded based on prior diagnostics. All items used 5-point Likert responses (1 = Strongly disagree to 5 = Strongly agree). Subscale scores were the mean of their items. Ethical Concern items were reverse-coded so that higher scores indicate fewer concerns and thus more favorable attitudes. A Total Attitudes score was computed as the mean of subscale means.

### Covariates

Prespecified covariates included age (years, continuous), gender (self-reported; reference specified in model tables), and education (ordered categories; reference specified). Self-rated health (1–5) and chronic condition (yes/no) were initially considered but not retained in the final adjusted models. Coding and reference categories are shown alongside model outputs.

### Item development and content validity

Item development followed a conceptual framework that integrated health literacy, digital information appraisal, and technology acceptance domains. An initial pool of 32 items was generated to ensure broad conceptual coverage across perceived benefit, intention to use, trust in information, and ethical concern. Content validity was established through a five-member multidisciplinary expert panel representing digital health, clinical sciences, medical informatics, ethics, and psychometrics. Experts evaluated each item for clarity, relevance, and representativeness, and items receiving low consensus or limited conceptual contribution were either revised or removed. Based on aggregated expert feedback, seven items were eliminated due to redundancy or insufficient relevance, and five items were reworded for improved clarity. Face validity was further assessed in a 20-participant pilot test in which individuals rated item clarity, readability, and perceived overlap; this process resulted in minor adjustments to simplify phrasing and enhance interpretability. The final 15-item version therefore reflected both expert consensus and end-user comprehension, ensuring that the retained items demonstrated adequate conceptual precision and practical understandability.

### Procedure and data quality

After consent, questionnaires were completed on site. Data were screened for eligibility, duplicates, straight-lining, and improbable completion times. Missingness was summarized per instrument. For regression analyses and descriptive summaries, low missingness permitted listwise deletion (<5%–10%). For CFA with WLSMV estimation, pairwise-present handling was applied automatically; effective N is reported in tables.

### Psychometric analyses

#### Assumption checks

Sampling adequacy and factorability were assessed via KMO and Bartlett's test. Both supported factor analysis (Table 1).

**Table 1:** KMO and bartlett's test of sphericity.

Statistic	Value	df	*p*
KMO (overall)	.945	—	—
Bartlett's *χ*^2^	4,404	91	<.001

Adequacy tests indicated suitability for factor analysis.

#### Exploratory factor analysis (EFA)

EFA was conducted to explore the latent structure underlying the initial item set and to determine whether theorized constructs emerged empirically. Minimum residual (minres) extraction with oblimin rotation was used, suppressing loadings <.30. A three-factor pattern appeared: Items 1–8 loaded together, Items 9–11 formed a second factor, and Items 13–15 formed a third. Factor correlations were strong, indicating the plausibility of a higher-order structure (Table 2).

**Table 2:** Pattern loadings and uniqueness (minres, oblimin; values <.30 suppressed).

Item	F1	F2	F3	Uniqueness
1	.714	—	—	.282
2	.915	—	—	.227
3	.798	—	—	.210
4	.846	—	—	.199
5	.568	.328	—	.309
6	.904	—	—	.182
7	.869	—	—	.238
8	.843	—	—	.185
9	—	.864	—	.146
10	—	.858	—	.155
11	—	.661	—	.189
13	—	—	.831	.268
14	—	—	.872	.208
15	—	—	.772	.277

#### Confirmatory factor analysis (CFA)

CFA tested whether the theorized four-factor structure—aligned with conceptual domains—fit the data better than a three-factor alternative suggested by EFA (merging Items 1–8). Competing models were evaluated to determine the most parsimonious yet theoretically coherent specification (Table 3).

**Table 3:** Model comparison: global fit indices.

Model	χ^2^ (df)	CFI	TLI	SRMR	RMSEA	90% CI RMSEA
Three-factor (Benefit + Intention merged)	348 (74)	0.940	0.926	0.034	0.110	0.099–0.122
Four-factor (second-order)	272 (72)	0.955	0.943	0.037	0.092	0.080–0.106

Although EFA suggested a unified structure for Items 1–8, the convergence between perceived benefit and intention to use reflects their well-documented conceptual proximity in technology acceptance research rather than true unidimensionality. CFA provides a more rigorous basis for testing these theoretically grounded distinctions by imposing measurement constraints and enabling direct comparison of competing structures (29, 30). In this study, retaining Benefit and Intention as distinct yet related factors improved fit and preserved theoretical interpretability. The RMSEA value for the four-factor model was in the upper-moderate range (RMSEA = .092; 90% CI = .080–.106), indicating borderline but still acceptable fit; such elevations are well documented in models with relatively few degrees of freedom and strong loadings, where RMSEA tends to overestimate misfit. Consistent with methodological recommendations emphasizing evaluation across multiple indices rather than strict RMSEA cutoffs (31–33), the constellation of CFI, TLI, SRMR, and standardized loadings collectively supported the adequacy of the four-factor higher-order structure, which was therefore retained as the primary measurement model.

### Reliability and validity

Internal consistency for CFA-retained items (excluding Item 12) was excellent: Cronbach's *α* (total) = .961. All subscales demonstrated excellent internal consistency (*α* = .897–.936; *ω* = .898–.937). Composite reliability values ranged from.898 to.937, and AVE values from.745 to.831, exceeding recommended thresholds (CR > .70; AVE >.50). Subscale intercorrelations were high (r = .558–.892). The Fornell–Larcker criterion was satisfied for most factor pairs; however, the Perceived Benefit–Intention pair slightly exceeded the square root of AVE (r = .892 vs. √AVE = .883–.884), indicating partial overlap. This pattern was consistent with the HTMT ratio (∼.96), which approached the conservative threshold (.85/.90), suggesting that these two constructs are closely related yet empirically distinguishable. Given the high association between these subscales, this partial redundancy should be further explored in future scale refinement, for example by revising overlapping item wording or testing higher-order or bifactor structures to strengthen discriminant validity.

### Outcomes

Primary outcomes were AI-KUAS subscale scores (Perceived Benefit, Intention to Use, Trust in Information, Ethical Concern) and the second-order Attitudes score. A Total Attitudes score (mean of subscale means, after reversal as required) served as a secondary summary.

### Statistical analysis (associations with health literacy)

Descriptive statistics summarized items and scale scores (means ± SD) and categorical variables (n, %). Primary analyses examined associations between health literacy (HLS-14-TR) and AI-KUAS outcomes:

1. Unadjusted Pearson/Spearman correlations
2. Adjusted multiple linear regression with AI-KUAS Total as the outcome and health literacy as the focal predictor, adjusting for age, gender, and education. We report standardized *β*, 95% CI, p, and R^2^.
3. Exploratory subscales: separate adjusted models for each subscale with Benjamini–Hochberg FDR (q = 0.05) across the four tests
4. Diagnostics: residual plots, heteroskedasticity tests (e.g., Breusch–Pagan), and HC3 robust SEs where indicated. VIF < 5 confirmed absence of problematic multicollinearity.
5. Sensitivity analyses: (i) alternative specification substituting a digital-literacy proxy (if available); (ii) non-linear terms (restricted cubic splines) when suggested by residual patterns; and (iii) models refit after winsorizing extreme values (1st/99th percentiles).

For group comparisons (age, education, occupation), we applied one-way ANOVA with Tukey's HSD *post-hoc* tests. Homogeneity of variances was inspected using Levene's test. Analyses were performed in jamovi (version 2.6.44) with lavaan back-end for CFA. Reliability was assessed using Cronbach's *α* and McDonald's *ω*, while convergent validity was examined through Composite Reliability (CR) and Average Variance Extracted (AVE). Discriminant validity was evaluated using the Fornell–Larcker criterion and the Heterotrait–Monotrait ratio (HTMT). Reliability and CR/AVE calculations were cross-checked in R, and all descriptive statistics, t-tests, ANOVAs, and *post-hoc* comparisons were conducted in IBM SPSS Statistics (version 29). Sensitivity analyses comprised winsorization (1st/99th percentiles) and polynomial extensions of the focal predictor (linear, quadratic, cubic). These checks were pre-specified and are reported alongside the main results.

### Missing data

For scale computation and regressions, we used listwise deletion after confirming low missingness (threshold <5%–10%). For CFA under WLSMV, the software's pairwise present handling was used. Each table reports analysis-specific N and missingness pattern.

## Results

Participants were predominantly young adults, with 42.5% aged 18–24 years, followed by 23.1% aged 35–44 years. The sample was composed of 59.0% females and 41.0% males. In terms of educational attainment, the majority held a bachelor's (36.3%) or associate degree (32.9%), with master's and doctoral degrees reported less frequently (16.6% and 7.1%, respectively). Daily use of AI systems was reported by only 10.9% of participants, whereas 28.1% indicated frequent use and 34.4% reported occasional use. Educational and research purposes were the most commonly cited reason for AI use (71.2%), followed by routine tasks (59.9%) and health information seeking (33.9%). ChatGPT emerged as the most widely used AI system (90.7%), whereas usage of alternatives such as Gemini, DeepSeek, and Claude was markedly lower (Table 4).

**Table 4:** Descriptive characteristics and AI system usage (application sample, *N* = 354; multiple responses allowed for AI purpose/system use).

Measure	Category/Value	n	%
Age	18–24	149	42.5
	25–34	65	18.5
	35–44	81	23.1
	45–54	41	11.7
	55+	15	4.3
Gender	Female	206	59.0
	Male	143	41.0
Education	High school	25	7.1
	Associate	115	32.9
	Bachelor's	127	36.3
	Master's	58	16.6
	Doctorate	25	7.1
AI use frequency	Daily	38	10.9
	Frequently	98	28.1
	Occasionally	120	34.4
	Rarely	74	21.2
	Never	19	5.4
Purpose of AI use	Education/Research	252	71.2
	Daily routine tasks	212	59.9
	Health information seeking	120	33.9
	Entertainment	99	28.0
	Other	80	22.6
AI system used	ChatGPT	321	90.7
	Gemini	70	19.8
	DeepSeek	60	16.9
	Grok	64	18.1
	Claude	19	5.4
	Bing AI	20	5.6
	Ada Health	21	5.9
	Buoy Health	19	5.4

Independent samples t-tests showed no statistically significant gender differences in attitudes toward AI or health literacy. Women reported marginally higher mean scores on both AI-KUAS (3.44 vs. 3.30) and HLS-14 (3.10 vs. 3.01), yet the differences were nonsignificant (*p* = .208 and *p* = .397). Effect sizes were negligible (Cohen's d = 0.14 for AI-KUAS and 0.09 for HLS-14), indicating that gender did not meaningfully contribute to variability in either outcome. Assumption checks confirmed acceptable normality and homogeneity of variances, with no indication of problematic outliers or unequal variances. Full descriptive and inferential statistics are presented in Table 5.

**Table 5:** Gender differences in AI-KUAS and HLS-14 scores (application sample, *N* = 349).

Measure	Reference (Lowest)	Higher Group (M ± SD)	*t*(df)	*p*	Mean diff (High−Ref)	95% CI [LL, UL]	Cohen's *d*
AI-KUAS	Men 3.30 (1.08)	Women 3.44 (0.98)	1.26 (347)	.208	0.14	[−0.08, 0.36]	0.14
HLS-14	Men 3.01 (0.88)	Women 3.10 (0.90)	0.85 (347)	.397	0.08	[−0.11, 0.27]	0.09

Cohen's d values are based on pooled standard deviations.

One-way ANOVA revealed significant differences across age groups for both AI attitudes and health literacy. For AI-KUAS total scores, the model was significant, F(4, 346) = 4.34, *p* = .002, and Tukey's HSD *post-hoc* tests showed that participants aged 25–34 and 35–44 scored higher than those aged 18–24 (mean differences = 0.51 and 0.40; Cohen's d = 0.52 and 0.39). A similar pattern appeared for health literacy, F(4, 346) = 9.70, *p* < .001, with the 35–44 and 45–54 groups scoring significantly higher than the youngest group (e.g., d = 0.58 and d = 0.89). These effects reflect a graded age-related pattern in both constructs. Assumption checks showed acceptable normality and homogeneity of variances (Levene's *p* > .05). Age categories followed standard demographic groupings used in Turkish population surveys. Full descriptive and inferential statistics are summarized in Table 6.

**Table 6:** Age group comparisons in AI-KUAS and HLS-14 scores (application sample, *N* = 351).

Measure	Reference (Lowest) (M ± SD)	Higher Group (M ± SD)	Test	*p*	Mean diff (High−Ref)	95% CI [LL, UL]	Cohen's d
AI-KUAS	18–24: 3.15 (1.04)	25–34: 3.66 (0.86)	F(4,346) = 4.34	.002	0.51	[0.10, 0.92]	0.52
		35–44: 3.55 (1.03)			0.40	[0.02, 0.78]	0.39
HLS-14	18–24: 2.77 (0.89)	25–34: 3.21 (0.76)	F(4,346) = 9.70	<.001	0.44	[0.09, 0.79]	0.52
		35–44: 3.27 (0.82)			0.51	[0.18, 0.83]	0.58
		45–54: 3.55 (0.85)			0.78	[0.37, 1.20]	0.89

Effect sizes (Cohen's d) are reported for pairwise *post-hoc* comparisons; values of 0.2, 0.5, and 0.8 are conventionally interpreted as small, medium, and large.

AI-KUAS and HLS-14 scores differed significantly by educational attainment. Participants with associate degrees showed the lowest mean scores (AI-KUAS = 3.22; HLS-14 = 2.85), whereas those with master's and doctoral degrees reported the highest values (AI-KUAS = 3.81 and 3.64; HLS-14 = 3.41 and 3.64, respectively). One-way ANOVA confirmed that these differences were statistically significant for both AI attitudes, F(4, 345) = 8.84, *p* < .001, and health literacy, F(4, 345) = 8.50, *p* < .001. Tukey's HSD *post-hoc* comparisons indicated medium-to-large effect sizes when postgraduate participants were compared with associate- or bachelor-level groups (AI-KUAS: d = 0.65–1.35; HLS-14: d = 0.82–1.07). These graded differences support a robust education-related gradient across both constructs. Assumption checks showed acceptable normality and homogeneity of variances (Levene's *p* > .05). Education categories followed standard Turkish higher-education classifications (associate, bachelor, master's, doctoral). Full numerical results are presented in Table 7.

**Table 7:** Education group comparisons in AI-KUAS and HLS-14 scores (application sample, *N* = 350).

Outcome	Group (M ± SD)	F(df)	*p*	Mean diff vs Ref	95% CI	Cohen's d
AI-KUAS	Assoc. (3.22 ± 1.08)	8.84 (4,345)	<.001	0.69	[0.10,1.29]	0.65
	Bachelor (3.43 ± 0.92)			0.91	[0.32,1.50]	0.91
	Master (3.81 ± 0.87)			1.29	[0.64,1.93]	1.35
	Doctorate (3.64 ± 0.94)			1.11	[0.35,1.87]	1.16
HLS-14	Assoc. (2.85 ± 0.89)	8.50 (4,345)	<.001	0.21	[−0.35,0.78]	0.19
	Bachelor (3.08 ± 0.82)			0.44	[−0.12,1.01]	0.39
	Master (3.41 ± 0.78)			0.77	[0.21,1.33]	0.82
	Doctorate (3.64 ± 0.81)			0.99	[0.33,1.66]	1.07

Effect sizes (Cohen's d) are reported for pairwise *post-hoc* comparisons; values of 0.2, 0.5, and 0.8 are conventionally interpreted as small, medium, and large.

AI-KUAS and HLS-14 scores differed significantly across occupational categories. Participants working in dental health exhibited the lowest mean scores on both measures (AI-KUAS = 2.00; HLS-14 = 2.30), whereas academic professionals reported the highest values (AI-KUAS = 3.92; HLS-14 = 3.76). One-way ANOVA confirmed statistically significant differences for both AI attitudes, F(10, 308) = 3.02, *p* = .001, and health literacy, F(10, 308) = 3.45, *p* < .001. Tukey's HSD *post-hoc* tests revealed particularly large effect sizes when comparing academic and clinical professionals with the dental group (Cohen's d > 1.50 for several contrasts), indicating substantial occupational disparities. These findings suggest that roles involving higher exposure to digital or clinical decision-support systems are associated with more positive AI attitudes and higher health literacy. Assumption checks indicated acceptable normality and homogeneity of variances (Levene's *p* > .05). Occupational categories were defined using standard Turkish workforce classifications, though unequal subgroup sizes should be considered when interpreting effect magnitudes. Full descriptive and inferential results appear in Table 8.

**Table 8:** Occupation group comparisons in AI-KUAS and HLS-14 scores (application sample, *N* = 319).

Measure	Reference (Lowest)	Higher Group (M ± SD)	*F*(df)	*p*	Mean diff (High−Ref)	95% CI [LL, UL]	Cohen's *d*
AI-KUAS	Dental health 2.00 (0.75)	Student 3.39 (0.94)	3.02 (10, 308)	.001	1.39	[0.25, 2.52]	1.57
		Teacher 3.56 (1.10)			1.56	[0.36, 2.76]	1.61
		Civil servant 3.64 (1.04)			1.64	[0.35, 2.93]	1.70
		Med. documentation 3.41 (0.91)			1.41	[0.18, 2.65]	1.62
		Academic 3.92 (0.87)			1.92	[0.65, 3.18]	2.20
		Doctor/Nurse 3.62 (1.05)			1.62	[0.33, 2.91]	1.69
		Consultancy 3.52 (0.88)			1.52	[0.12, 2.91]	1.72
		Other 3.22 (1.06)			1.22	[0.08, 2.36]	1.34
HLS-14	Dental health 2.30 (0.86)	Academic 3.76 (0.89)	3.45 (10, 308)	<.001	1.46	[0.37, 2.55]	1.65
		Med. documentation 2.90 (0.65)			0.86	[0.08, 1.63]	1.06
		Student 2.86 (0.90)			0.89	[0.23, 1.55]	1.08
		Doctor/Nurse 3.04 (0.90)			0.74	[0.07, 1.56]	0.88

A Pearson correlation analysis indicated a statistically significant and moderately strong positive relationship between AI attitudes and health literacy (*r* = .51, *p* < .001). This association suggests that individuals with higher health literacy are more likely to report positive attitudes toward AI use in health contexts. The effect size is practically meaningful (Table 9).

**Table 9:** Correlation between AI-KUAS and HLS-14 (application sample, *N* = 354).

Variables	1	2
1. AI-KUAS	—	.51**
2. HLS-14	.51**	—

*N* = 354. *p* < .01 (two-tailed).

Positive and significant correlations emerged between health literacy and both the AI-KUAS total score and its subscales. Instead of reporting simple regressions, we present adjusted multiple regression models. These models indicate that health literacy remains strongly associated with attitudes toward AI after controlling for age, gender, and education. Health literacy was significantly associated with all outcomes (*β* = .39–.50, *p* < .001). The largest standardized association was observed for Perceived Benefit (*β* = .503), whereas the smallest—though still statistically significant—was for Trust in Information (*β* = .385). Education was independently associated with higher scores on the Total, Benefit, Intention, and Ethical Concern subscales (*β* = .16–.19, *p* ≤ .002). Neither age nor gender reached significance in any model. Explained variance ranged from.17 to.31, with the total attitudes model accounting for 30% of the variance (Table 10). All associations remained significant after Benjamini–Hochberg correction (q = .05). Sensitivity analyses using winsorization (1st/99th percentiles) produced virtually identical estimates. Adding polynomial terms for health literacy (linear, quadratic, cubic) did not materially improve model fit (R^2^ ∼.23–.31) and did not alter the substantive conclusions; we therefore retained the parsimonious linear specification.

**Table 10:** Multiple regression of health literacy predicting attitudes toward AI (*N* = 354).

Outcome	Predictor	*β*	95% CI	*p*	R^2^
Total AI-KUAS	Health literacy	.488	[.394,.583]	<.001	.30
	Age	-.065	[-.165,.034]	.198	
	Gender	-.047	[-.138,.044]	.309	
	Education	.168	[.069,.268]	.001	
Perceived Benefit	Health literacy	.503	[.409,.597]	<.001	.31
	Age	-.092	[-.191,.007]	.070	
	Gender	-.052	[-.143,.039]	.262	
	Education	.156	[.057,.255]	.002	
Intention to Use	Health literacy	.418	[.319,.517]	<.001	.23
	Age	-.049	[-.154,.055]	.356	
	Gender	-.013	[-.108,.083]	.795	
	Education	.162	[.058,.267]	.002	
Trust in Information	Health literacy	.385	[.283,.488]	<.001	.17
	Age	-.022	[-.131,.086]	.688	
	Gender	-.047	[-.146,.053]	.356	
	Education	.078	[-.030,.186]	.155	
Ethical Concern	Health literacy	.409	[.310,.507]	<.001	.24
	Age	-.062	[-.165,.042]	.245	
	Gender	-.063	[-.158,.032]	.192	
	Education	.191	[.087,.295]	<.001	

## Discussion

The findings are consistent with health literacy as a proximal correlate of more favorable attitudes toward clinical AI. Correlation and regression estimates from the application sample suggest that health literacy is better interpreted as a near-term correlate rather than a remote background trait. Recent syntheses show that higher digital or general health literacy aligns with more effective navigation of online health information, stronger intention to use digital tools, and greater persistence in technology-supported self-management, consistent with the present pattern of associations (6, 12, 34). Large-scale surveys on clinical AI also indicate that users value disclosure and oversight, directing attention to implementation practices that support safer participation (35).

In our adjusted models, age did not reach significance; any apparent crude gradients attenuated after accounting for education and health literacy, aligning with evidence that cohort effects on digital health literacy are largely mediated by exposure and education (7, 34). Studies in older or underserved populations show that digital health literacy can lag without targeted support, although repeated interactions with services and skill-building reduce gaps over time (36). Measurement choices likely shape observed gradients, since self-report scales capture perceived capability while task-based items are closer to realized behaviors (6, 37).

Gender differences were negligible. Systematic reviews report that sex is not a stable determinant of digital health literacy after adjusting for education and exposure, which is consistent with the present null findings and small effect sizes (6, 7). Sector-specific surveys sometimes report higher AI engagement among men or greater AI anxiety among women, yet these differences attenuate when prior technology use and task demands are modeled (38).

Education and occupation exhibited graded associations with both outcomes, with academics and clinical professionals scoring highest. Prior evidence links higher educational attainment and domain exposure to stronger navigation of online health information, more frequent patient-portal use, and more favorable attitudes toward AI (7, 12, 37, 39). Workforce and service-design reports emphasize that familiarity with decision-support tools and routine contact with digital workflows reinforce learned efficacy and trust, which plausibly explains the present occupational contrasts (40, 41).

Trust and acceptance are shaped by design choices as much as by user attributes. Reviews of explainable AI in clinical settings converge on the conclusion that user-comprehensible rationales, calibrated uncertainty communication, and clear role delineation for human oversight can improve confidence without inflating unwarranted certainty (19, 21, 42). Empirical work likewise shows that disclosure of AI use and accountability cues matter for acceptance at the point of care (43, 44). Transparency about development, testing, and known limitations remains uneven across products, which sustains variability in trust despite strong model performance claims (45).

Policy guidance reinforces these implementation levers. The World Health Organization recommends literacy-sensitive communication, notification of AI use, and evaluation plans that monitor equity effects over time (41). Recent OECD analyses emphasize operationalising trustworthy AI through workforce preparation, measurement frameworks, and safeguards that prevent amplification of inequities during scaling (40). These recommendations are consistent with our findings linking higher literacy and professional exposure with more favorable attitudes, and they point to modifiable levers that may support engagement among less advantaged groups.

In practical terms, the findings suggest that health-literate users interpret AI-generated health information more effectively, identify when clinical follow-up is needed, and evaluate AI advice against prior medical knowledge rather than accepting it at face value. Higher literacy was associated with stronger perceived benefit and greater trust in AI outputs, indicating that individuals with better appraisal and navigation skills are more capable of distinguishing actionable insights from uncertainty or algorithmic limitations. This pattern aligns with evidence showing that people with higher literacy make fewer misinterpretations of symptom-checker advice, engage more safely with digital triage systems, and use patient portals more appropriately for decision-making. Taken together, the results imply that literacy-sensitive communication and onboarding could reduce misinterpretations, prevent over-reliance on AI, and support safer integration of AI into clinical encounters.

Future research should extend these findings in several ways. First, longitudinal and experimental designs are needed to determine whether improving health literacy—or targeted components such as appraisal skills—causally increases appropriate trust and safer engagement with clinical AI–assisted care. Trials that test literacy-aligned onboarding, layered explanations, or uncertainty-aware communication could clarify which explanation formats most effectively support users with varying literacy levels. Second, studies should pair self-reported attitudes with behavioral measures, such as navigation logs, triage decisions, or clinical follow-up after interacting with AI tools, to capture how literacy shapes real-world use rather than stated preferences alone. Third, measurement invariance across age, gender, education, and professional groups should be systematically evaluated before subgroup comparisons are generalized. Relatedly, the very high internal consistency observed here (*α* = .961) supports reliability, while also leaving open the possibility that some items overlap in content; future work could examine redundancy and, if warranted, streamline the item set without narrowing its conceptual coverage. Finally, diverse clinical settings—including imaging, triage, primary care portals, and chronic disease management platforms—should be examined to determine how literacy interacts with contextual factors such as task complexity, clinician oversight, and risk level. These directions would enable more precise theorizing about literacy-AI mechanisms and inform scalable, equity-oriented implementation strategies.

Several limitations qualify these interpretations. The cross-sectional design supports association rather than causation, leaving residual confounding by socioeconomic status, numeracy, and prior technology exposure as plausible alternatives. Our outcomes are self-reported attitudes, not logged behaviors; thus, results speak to willingness and trust rather than actual use at clinical touchpoints (9, 46). We did not test measurement invariance across demographic groups; therefore, mean comparisons by gender, age, education, or occupation should be interpreted cautiously until configural/metric/scalar invariance is demonstrated. The application sample was skewed toward young adults (e.g., 42.5% aged 18–24) and heavily exposed to a single general-purpose model (e.g., 90.7% ChatGPT), which may limit generalisability to settings where clinical AI is accessed through portals, imaging suites, or triage tools rather than chat interfaces; the non-probability recruitment strategy (purposive district selection and convenience sampling in public venues) may also have introduced selection effects that constrain broader population inference. Listwise deletion of missing data and reliance on a single instrument set motivate replication with alternative estimators, sensitivity analyses, and pre-registered model specifications. Model fit also warrants caution: while CFI (.955), TLI (.943), and SRMR (.037) were within acceptable ranges, RMSEA was.092. Because RMSEA is sensitive to degrees of freedom and minor model misspecification and can over-reject otherwise adequate models when df are modest (df = 72), we interpreted fit holistically rather than privileging a single index. The strong CFI/TLI and low SRMR indicate good reproduction of the observed covariance structure, whereas the RMSEA value suggests residual misfit that should be revisited in independent samples and, where theoretically justified, under alternative specifications. Accordingly, we interpreted fit holistically using multiple indices rather than a single cutoff (32, 33).

## Data Availability

The original contributions presented in the study are included in the article/Supplementary Material, further inquiries can be directed to the corresponding author.
