# Supplementary material for: Health literacy and attitudes toward clinical AI: a cross-sectional study in Istanbul
Source: Front Digit Health. 2026 May 7;8:1771286. doi: 10.3389/fdgth.2026.1771286 (PMC13190160; doi:10.3389/fdgth.2026.1771286)
Supplement: Supplementary file 1 [file Datasheet1.docx]

**Supplementary File 1**

**Artificial Intelligence Knowledge, Usage, and Attitudes Scale (AI-KUAS) English Version**

**Administration & Scoring**

**Administration & Scale**

- Adults (18+), self-administered.
- Response options for all items: 1 = Strongly disagree … 5 = Strongly agree.
- Analysis version: 14 items.
- Subscales:
  - Perceived Benefit (1–4)
  - Intention to Use (5–8)
  - Trust in Information (9–11)
  - Ethical Concern (12–14)
- Scoring: Subscales = mean of items; Total Attitudes = mean of four subscale means.
- Reverse-score: 12, 13, 14 (score as 6 − response).

**Item Content (by subscale)**

**Perceived Benefit (1–4)**

1. AI systems allow me to access health-related information more quickly.
2. AI-based tools make my daily life easier.
3. The suggestions provided by AI systems support my personal decisions.
4. AI-supported solutions are more practical than traditional sources of information.

**Intention to Use (5–8)**

5. I actively use AI systems in my daily life.
6. I intend to use more AI-supported applications in the future.
7. Using AI tools is becoming a habit for me.
8. I am willing to try new AI-based applications when they become available.

**Trust in Information (9–11)**

9. I largely trust the health information provided by AI systems.
10. AI-generated answers are generally correct and based on scientific evidence.
11. I feel comfortable relying on information from AI systems when making health-related decisions.

**Ethical Concern (12–14) — reverse-score all**

12. I think AI may threaten individuals’ privacy.
13. AI-supported tools could reduce human control in the future.
14. I worry that AI-based systems might produce inaccurate or misleading information.

**Citation:**
Sönmez K. *Health Literacy and Attitudes Toward Clinical AI: A Cross-Sectional Study in Istanbul*, 2025. Instrument developed by the author for this study.
